# Sonographic prediction of intraductal papillary carcinoma with partially cystic breast lesions

**DOI:** 10.1186/s12880-022-00934-y

**Published:** 2023-01-06

**Authors:** Luying Gao, Xingjian Lai, Jing Zhang, Yuxin Jiang, Jianchu Li

**Affiliations:** Department of Ultrasound, Peking Union Medical College Hospital, Chinese Academy of Medical Sciences & Peking Union Medical College, 9 Dongdansantiao, Beijing, 100730 China

**Keywords:** Breast cancer, Intraductal papillary carcinoma, Intraductal papilloma, Ultrasound, Partially cystic breast lesion

## Abstract

**Background:**

Intraductal papillary carcinoma (IDPC) is a rare but fatal disease. Preoperative ultrasound diagnosis of IDPC remains challenging and meaningful. The aim of the study was to determine an effective ultrasound model to predict intraductal papillary carcinoma (IDPC) in patients with partially cystic breast lesions on ultrasound.

**Methods:**

We reviewed female patients with breast nodules who underwent biopsy or surgery between 2004 and 2019, and pathological results were used as the reference standard. We finally included 21 IDPC patients with partially cystic lesions on preoperative ultrasound matched to 40 patients with intraductal papilloma. The association of ultrasound features with IDPC was analysed.

**Results:**

Posterior echo enhancement (*P* < 0.001), tumour size (*P* = 0.002), irregular shape (*P* = 0.003), wide base (*P* = 0.003), solid-mainly component (*P* = 0.013), rich Doppler flow (*P* < 0.001) and multiple lesions (*P* = 0.044) were associated with IDPC by univariate analysis. Based on univariate analysis, variables were included in the regression analysis to obtain independent factors. The regression analysis showed that microcalcification, multiple lesions, posterior echo enhancement, wide base of solid components and rich colour Doppler flow were predictors for IDPC (*P* < 0.001). The collective model of the independent factors (microcalcification, multiple lesions, posterior echo enhancement, wide base of solid components and rich colour Doppler flow) could predict IDPC with an area under the curve (AUC) of 0.99 (95% CI 0.95–1.00). The collective model had a better net benefit demonstrated by the decision curve.

**Conclusion:**

Ultrasonic features may be an applicable model for predicting IDPC with partially cystic breast lesions on ultrasound and has a better potential to facilitate decision-making preoperatively.

## Background

Breast cancer incidence has increased during the past 2 decades [1]. Intraductal papillary carcinoma (IDPC) is rare, accounting for < 2–5% of breast cancer cases. Although the sample size of malignant ductal breast lesions is very narrow, identify patients with a high likelihood of IDPC ability could develop a more accurate surgical planning and reduce potential risks to patients and the overall health care costs.

Ultrasound (US) because of its cost-effectiveness, non-radiation and high sensitivity is widely used for prediction of breast cancer [2]. In the assessment of intraductal masses, US is more specific than mammography and magnetic resonance imaging (MRI), and may be considered as the selective manner [3–6]. However, intraductal masses lack the classical ultrasound features of malignancy, such as taller-than-wide shape, and irregular margins (infiltrative, microlobulated). IDPC has some similarities with other intraductal breast masses in imaging and clinical manifestations, especially for those with partially cystic lesions on ultrasound, which is difficult to diagnose clinically. For the breast imaging reporting and data system (BI-RADS) system, intraductal lesions are one of the “special cases”. It is unclear whether all detected by US should be classified as BI-RADS 4a because there is no clear clinical and radiological prediction of malignancy. Whether biopsy is needed for all intraductal masses is still in debate [7, 8]. Moreover, there are possibilities of false-negative results from biopsy procedures, and patients may experience inadequate resection in the first operation.

To the best of our knowledge, none of the studies has evaluated the US features of IDPC with partially cystic lesions. Except for intraductal papillary carcinoma (IDPC), there are previous papers analysing the ultrasound features of other malignant lesions of intraductal masses (including ductal carcinoma in situ and invasive ductal carcinoma). These ultrasound features include filling the duct more completely and involving more branch ducts [7, 9]. Herein, we evaluated the US features of IDPC with the pathological results obtained after surgery, aiming to explore an effective ultrasound model of identifying intraductal papillary carcinoma with partially cystic lesions on US preoperatively.

## Methods

### Patients

We retrospectively studied female patients with breast nodules who underwent excisional biopsy or surgery between January 2004 and October 2019 at our centre. The final pathologic results were considered the diagnostic gold standard. A total of 99 patients were diagnosed with intraductal papillary carcinoma based on pathological examination with WHO classification in our centre. The following criteria were applied: (1) partially cystic lesions on preoperative ultrasound (solid lesions were excluded, N = 69); (2) patients younger than 18 years of age were excluded (N = 1); (3) patients who received treatments before surgery were excluded (N = 2); and (4) patients who had a history of breast cancer in the previous study were removed (N = 6). A total of 21 lesions from 21 patients were included. Using the same database who underwent excisional biopsy or surgery during the same period, patients with intraductal papilloma were enrolled as a control group. Patients who met the exclusion criteria were excluded. An age-matched control group comprising 40 lesions from 40 patients was randomly selected.

### Breast US examination

All US examinations were performed with Phillips HDI 5000, IU 22 (Philips Healthcare, Eindhoven, Netherlands), GE Logiq 9 or Logiq 7 (GE Healthcare, Milwaukee, WI, USA) devices equipped with either a 5–12 MHz or an 8–15 MHz linear-array transducer. US was performed by radiologists in our centre before surgery or biopsy, and US images were retrospectively evaluated by two radiologists who were experienced in breast US and were blinded to the patient clinical data for the study (staff radiologists with 8 and 12 years of experience). In cases involving a discrepancy between the assessments, a consensus was reached after discussion. The tumour size, tumour shape of solid component, single/multiple lesions, echogenicity of solid component, component (cyst-mainly or solid-mainly), homogeneity (homogeneity or heterogeneity), base of solid components (wide-basement or acute angle), presence of duct ectasia, posterior echo enhancement and microcalcifications were evaluated by B-mode US. Vascularity was classified as 4 patterns (no flow, minimal, moderate, or marked) by colour Doppler flow [10]. In cases involving a discrepancy between the assessments, a consensus was reached after discussion.

### Statistical analysis

Categorical variables were presented as frequencies, and analyzed using the chi-squared test. Quantitative data were presented as the mean ± standard deviation (SD), and analyzed using the Mann–Whitney U test or the unpaired t-test. The sensitivity, specificity, positive predictive value (PPV), negative predictive value (NPV) and accuracy was calculated according to the pathological findings. Cutoff value of the model was determined according to the ROC (receiver operating characteristic). Based on the parameters from the statistically significant results of univariate analysis, a multivariate logistic regression model was established. Decision curve analysis of the case–control study was carried out to compare the clinical applicability of different models. All statistical analyses were performed with SPSS software version 19.0 (Chicago, IL, USA) and R version 3.5.0 (R Foundation for Statistical Computing, www.R-project.org). Differences with *P* < 0.05 were considered statistically significant.

## Result

### Sonographic features of PTC patients with partially cystic intraductal papillary carcinoma

**Fig. 1:**
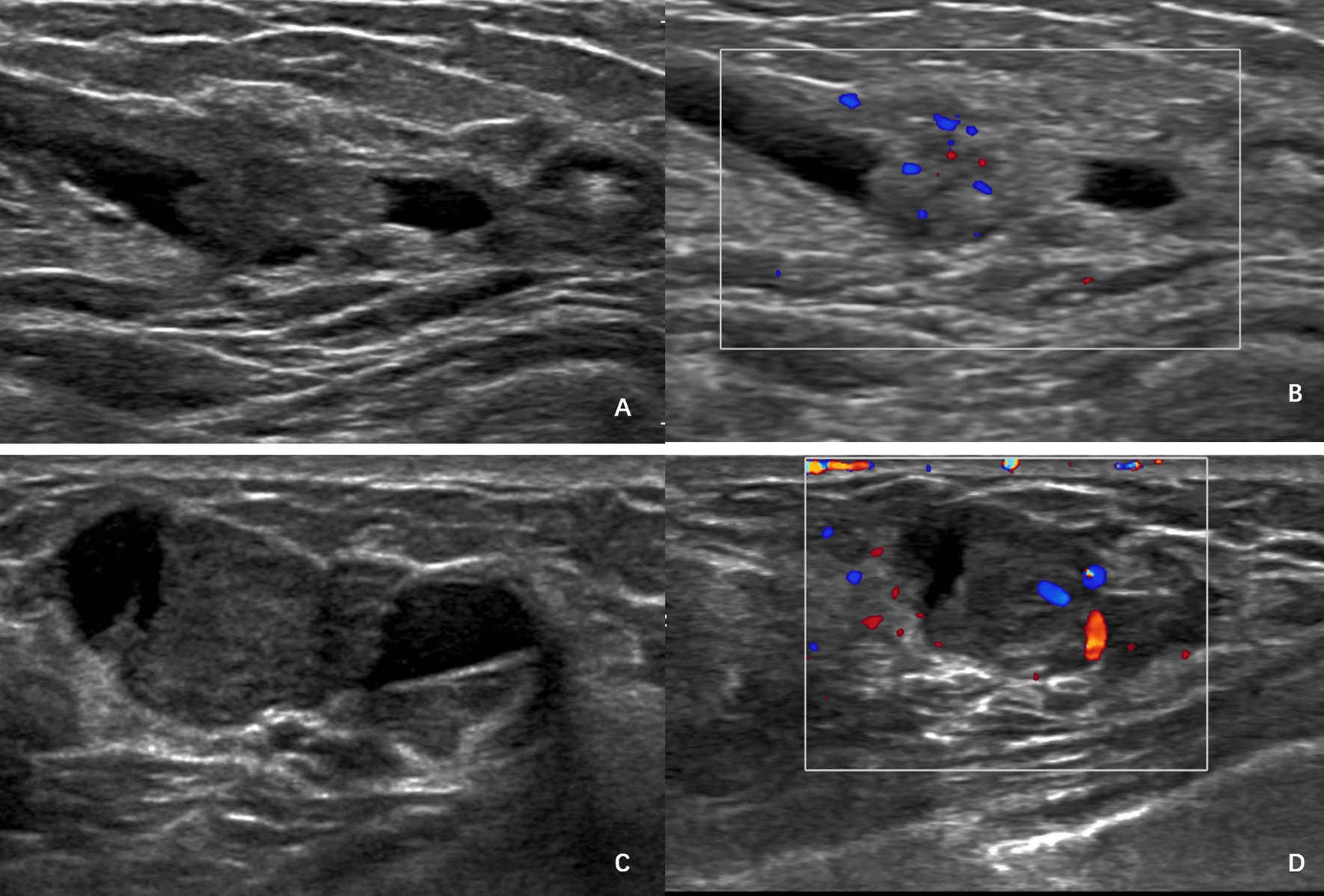
A breast masse from a young woman with intraductal papilloma. B-mode ultrasound showed a 2.0-cm narrow-base hypoechoic mass. The colour Doppler ultrasound image of the same mass reveals internal and peripheral blood flow signals (**A**,**B**). A breast masse from a young woman with intraductal papillary carcinoma, B-mode ultrasound showed a 0.9-cm wide-base hypoechoic mass. The colour Doppler ultrasound image of the same mass reveals internal and peripheral blood flow signals (**C**,**D**)

The correlation of US and clinical features between partially cystic IDPC and partially cystic intraductal papilloma is shown in Table 1. There were significant differences in tumour size (*P* = 0.002), tumour shape of solid components (*P* = 0.003), single/multiple lesions (*P* = 0.044), posterior echo enhancement (*P* < 0.001), component (*P* = 0.013), wide base of solid components (*P* = 0.003) and colour Doppler flow (*P* < 0.001) between the malignant and benign groups [Fig. 1]. However, microcalcification, homogeneity, echogenicity, and the presence of duct ectasia were not associated with IDPC (*P* > 0.05) [Table 1].

**Table 1:** Clinical and ultrasound characteristics

	Intraductal papilloma	Intraductal papillary carcinoma	Total	*P* value
Age (mean ± SD, years)	46.45 ± 12.16	52.95 ± 15.21	48.69 ± 13.53	0.10
Tumour size (mean ± SD, cm)	1.34 ± 0.89	2.24 ± 0.88	1.65 ± 0.98	< 0.001
*Tumour size (cm)*				
< 2	35 (87.5)	11 (52.4)	46	0.002
≥ 2	5 (12.5)	10 (47.6)	15	
*Tumour shape of solid components*				0.003
Regular	29 (72.5)	7 (33.3)	36	
Irregular	11 (27.5)	14 (66.7)	25	
*Lesions*				0.044
Single	15 (37.5)	2 (9.5)	17	
Multiple	25 (62.5)	19 (90.5)	44	
*Posterior echo enhancement*				< 0.001
Yes	15 (37.5)	20 (95.2)	35	
NO	25 (62.5)	1 (4.8)	26	
*Microcalcification*				0.11
Yes	1 (2.5)	3 (14.3)	4	
NO	39 (97.5)	18 (85.7)	57	
*Homogeneity*				0.11
Homogeneity	40 (100.0)	19 (90.5)	59	
Heterogeneity	0	2 (9.5)	2	
*Echogenicity*				0.18
Hypoechoic	37 (92.5)	17 (81.0)	54	
Complex	3 (7.5)	4 (19.0)	7	
*Component*				0.013
Solid-mainly	36 (90.0)	13 (61.9)	49	
Cyst-mainly	4 (10.0)	8 (38.1)	12	
*Wide base*				< 0.001
Yes	1 (2.5)	19 (90.5)	21	
NO	39 (97.5)	2 (9.5)	41	
*Presence of duct ectasia*				0.44
Yes	9 (22.5)	3 (14.3)	12	
NO	31 (77.5)	18 (85.7)	51	
*CDFI level*				0.041
0–1	31 (77.5)	9 (42.9)	40	
2–3	9 (22.5)	12 (57.1)	21	

*CDFI* colour Doppler flow imaging

### Diagnostic performance of the ultrasound characteristics

Among all the sonographic factors, receiver operating curve (ROC) analysis showed that posterior echo enhancement, tumour size (> 2 cm), irregular shape, wide base, and CDFI level are significant predictive factors for the diagnosis of IDPC. For the diagnosis of IDPC, the sensitivity, specificity, PPV, NPV, accuracy and AUC of posterior echo enhancement were 95.2%, 62.5%, 57.1%, 96.2%, 73.7% and 0.79 (95% CI 0.68–0.89), respectively. The sensitivity, specificity, PPV, NPV, accuracy and AUC of tumour size (> 2 cm) were 47.6%, 87.5%, 66.7%, 87.5%, 73.8% and 0.68 (95% CI 0.53–0.83), respectively. The sensitivity, specificity, PPV, NPV, accuracy and AUC of the irregular shape of solid components were 66.7%, 72.5%, 56.0%, 80.6%, 70.5% and 0.70 (95% CI 0.55–0.84), respectively. The sensitivity, specificity, PPV, NPV, accuracy and AUC of wide base solid components were 90.5%, 97.5%, 95.0%, 95.1%, 95.1% and 0.94 (95% CI 0.86–1.00), respectively. The sensitivity, specificity, PPV, NPV, accuracy and AUC of the CDFI level were 57.1%, 77.5%, 57.1%, 77.5%, 70.5% and 0.67 (95% CI 0.53–0.82), respectively [Table 2].

**Table 2:** Diagnostic efficiency of the ultrasound characteristics for intraductal papillary carcinoma

	Sensitivity (%)	Specificity (%)	PPV (%)	NPV (%)	Accuracy (%)	AUC
Posterior echo enhancement	95.2	62.5	57.1	96.2	73.7	0.79 (0.68–0.89)
Size (> 2 cm)	47.6	87.5	66.7	87.5	73.8	0.68 (0.53–0.83)
Irregular shape	66.7	72.5	56.0	80.6	70.5	0.70 (0.55–0.84)
Wide base	90.5	97.5	95.0	95.1	95.1	0.94 (0.86–1.00)
CDFI level 2–3	57.1	77.5	57.1	77.5	70.5	0.67 (0.53–0.82)

*CDFI* colour Doppler flow imaging

### *Diagnostic performance of the* sonographic prediction of partially cystic intraductal papillary carcinoma

Based on univariate analysis, variables were included in the logistic regression analysis. Microcalcification, multiple lesions, posterior echo enhancement, wide base of solid components and rich colour Doppler flow were found to be independent factors. The malignant group was more likely to have tumours with microcalcification (X_1,_ *P* < 0.001), posterior echo enhancement (X_2,_ *P* < 0.001), wide base of solid components (X_3,_ *P* < 0.001) and rich colour Doppler flow (X_4,_ *P* < 0.001), and multiple lesions (X_5,_ *P* < 0.001). The multivariate regression model was established as follows: Y = -69.05 + 18.61 * X_1_ + 17.01 * X_2_ + 52.73 * X_3_ + 17.91 * X_4_ + 33.43 * X_5_.

**Fig. 2:**
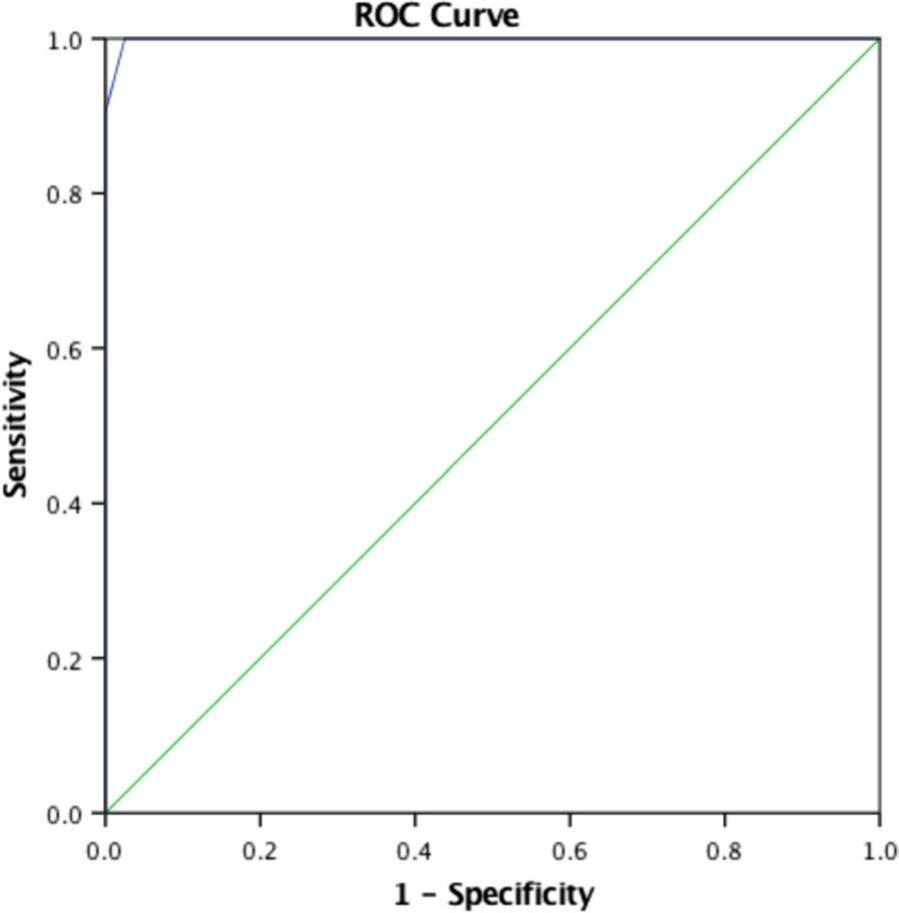
Receiver operating curve (ROC) analysis for the prediction models of intraductal papillary carcinoma

**Fig. 3:**
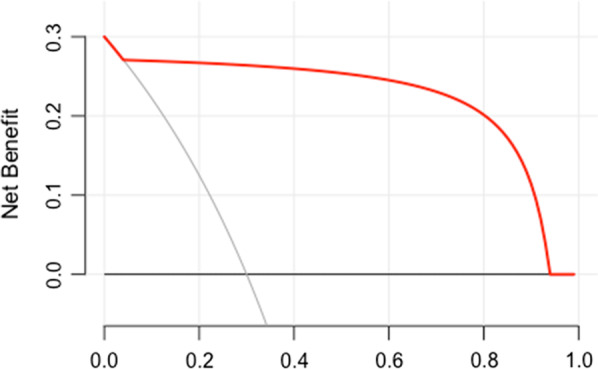
Decision curve analysis of the collective model including risk factors for microcalcification, multiple lesions, posterior echo enhancement, wide base of solid components and rich colour Doppler flow

Factors including microcalcification, multiple lesions, posterior echo enhancement, wide base of solid components and rich colour Doppler flow were finally involved in the collective model. The collective model could predict malignancy with an AUC of 0.99 (95% CI 0.95–1.00) [Fig. 2]. The decision curve showed that the collective model had a better net benefit with a wider range of threshold probabilities [Fig. 3].

## Discussion

In this study, aiming to analyse the ultrasound features of IDPC lesions, we compared the US features between IDPC and intraductal papilloma. A collective model (including microcalcification, multiple lesions, posterior echo enhancement, wide base of solid components and rich colour Doppler flow) was proposed as a reference for patient diagnosis. The collective model could predict malignancy with an AUC of 0.99 (95% CI 0.95–1.00). The collective model had a better net benefit demonstrated by the decision curve.

On the basis of the results of the present study, the partially cystic IDPC showed several differences from the known malignant sonographic features of solid breast nodules [11]. The findings of this study showed that hypoechogenicity was observed in 37 (92.5%) benign and 17 (81.0%) malignant groups. Therefore, hypoechogenicity was considered unhelpful in the differentiation of partially cystic IDPC from benign IDPC. In the study, microcalcifications and rich colour Doppler flow within the solid component of partly cystic lesions are considered the US features that raise the likelihood of IDPC. 3/21 IDPC had microcalcification on breast sonography, and there was only one microcalcification in the intraductal papilloma group. Twelve of 21 IDPCs had rich colour Doppler flow on breast sonography, and 9/40 with intraductal papilloma had rich colour Doppler flow. Similarly, colour Doppler ultrasound and microcalcifications are helpful for distinguishing between benign and malignant breast lesions [12, 13]. In BI-RADS Fifth Edition, microcalcification and colour Doppler flow imaging features are also important US characteristics of breast lesions to consider when stratification a nodule [11]. Since none of the studies has evaluated the US features of IDPC with partially cystic lesions, we found that traditional US features, such as microcalcifications and rich colour Doppler flow, are still helpful.

For diagnosing IDPC, the sensitivity, specificity, PPV, NPV, accuracy and AUC of a wide base of solid components were 90.5%, 97.5%, 95.0%, 95.1%, 95.1% and 0.94 (95% CI 0.86–1.00), respectively, which indicated good diagnostic value. Previous studies showed that the solid component of papilloma usually presents as a focal mass arising from the ductal wall, with a relatively narrow base for attachment [10, 14]. In our study, the component of intraductal papilloma was identified to have a narrow base arising from the wall. The wide or narrow base configuration means an acute angle or blunt angle, depending on the angle degree between the solid component and the adjacent cyst wall on real-time sonography. A previous study also showed that malignant intraductal masses tended to fill the duct more completely [7]. In total, 19/21 IDPCs had a wide base of the solid component on US, but only one lesion (1/40) had a wide base in the intraductal papilloma group. This may be due to the intracystic components of carcinomas requiring multiple vascular poles emerging from the base of the papillary projections [15].

In a previous study, posterior acoustic shadowing of a solid mass was suggestive of invasive carcinoma, but this feature is not a reliable indicator for malignancy [11, 14]. Unlike in these retrospective studies, the results of this study showed that posterior echo enhancement of a partially cystic mass increased the risk of IDPC. To our knowledge, there has been no other study on the diagnostic efficacy of posterior echo shadowing for partially cystic IDPC. This result may be explained by the fact that compared with the surrounding normal tissue, some tumours have a uniform internal structure. When close to the normal tissue, the boundary of the tissue is obvious, so repeated reflections occur, and the posterior echo is enhanced.

Malignancy rates of complex cystic and solid breast lesions contain 0.3%–50%, as reported in previous studies [16]. A range of malignant pathologic results has been detected, and the most common malignancies include intraductal carcinoma in situ (DCIS), invasive ductal carcinoma (IDC), intraductal papillary carcinoma (IDPC) [7, 16]. US appearance of DCIS are nonspecific. Calcified DCIS most commonly manifests as echogenic foci located within a mass or duct, associated with internal microlobulations, or distributed in a branch pattern. Noncalcified DCIS may manifest as a hypoechoic mass with microlobulated margins and no posterior acoustic features, or it may have a “pseudomicrocystic” appearance. Harmonic imaging and coronal reconstruction may improve the detection of noncalcified DCIS [17]. The typical US appearance of IDC was irregular hypoechoic lesions with microcalcification, and it may have posterior echo attenuation [18].

There are several limitations to our study. First, different radiologists performed the primary ultrasound examinations, which may lead to interobserver differences. Second, our study included a small number of patients with intraductal papillary carcinoma, and further studies with a larger patient population and prospective inclusion of intraductal masses with subjective criteria are needed to confirm our findings. Finally, only colour Doppler sonography was used to evaluate the vascularity of breast lesions.

## Conclusions

The ultrasound characteristics of partially cystic IDPC lesions included microcalcification, multiple lesions, posterior echo enhancement, wide base of solid components and rich colour Doppler flow. A collective model of all combinations of was highly reliable and careful correlation in terms of these sonographic features is needed. With improved technology, recognizing the US features of DCIS will become increasingly important for the detection of early-stage breast cancer.

## Data Availability

The datasets used and/or analyzed during the current study are available from the corresponding author on reasonable request.

## Abbreviations

CDFI: Colour Doppler flow imaging
ROC: Receiver operating curve
IDPC: Intraductal papillary carcinoma
BI-RADS: Breast imaging reporting and data system
MRI: Magnetic resonance imaging
US: Ultrasound
AUC: Area under the curve
PPV: Positive predictive value
NPV: Negative predictive value
SD: Standard deviation
